# Transmission-based association mapping of triglyceride levels in a longitudinal framework using quasi-likelihood

**DOI:** 10.1186/s12919-018-0147-4

**Published:** 2018-09-17

**Authors:** Hemant Kulkarni, Indranil Mukhopadhyay, Saurabh Ghosh

**Affiliations:** Human Genetics Unit, Indian Statistical Institute, 203 BT Road, Kolkata, India

## Abstract

Complex genetic traits are often characterized by multiple quantitative phenotypes. Because values of such phenotypes vary over time, it is thought that analyses of longitudinal data on the phenotypes may lead to increased power in detecting genetic association. In this paper, we extend a transmission-based association test applying quasi-likelihood that has been developed by us to the longitudinal framework and to carry out a genome-wide association analysis of triglyceride levels based on the data provided in GAW20. We consider different phenotype definitions based on administration of fenofibrate and obtain significant association findings within genes involved in heart diseases.

## Background

Most clinical end-point traits are governed by quantitative precursors and it may be a prudent strategy to analyze these precursor phenotypes for association mapping of a clinical end-point trait. The family-based design for detecting association as implemented in the classical transmission disequilibrium test [1] is a popular alternative to population-based case-control studies as it circumvents the problem of population stratification. We have developed a modification of a transmission-based test for quantitative traits proposed by us [2] by incorporating transmission information from both parents [3], instead of only the heterozygous parent in a family, based on the paradigm that the phenotype of an offspring is a function of the alleles transmitted by both parents. We adopt a quasi-likelihood approach [4] to develop a novel test statistic for association in the presence of linkage between a single-nucleotide polymorphism (SNP) and a quantitative trait. Although most association analyses are based on phenotype measured at single time points, longitudinal data on phenotypes carry more information on trait variation compared to cross-sectional data. However, the major statistical challenge in the association analyses of longitudinal phenotypes lies in the modeling of phenotype values over different time points. We extend our proposed method [3] in a longitudinal framework, and apply it to analyze triglyceride levels in families using data over the 4 time points provided in GAW20. We compare the association results for triglyceride levels based on transmission information from both parents with those based on transmission information only from heterozygous parents. We also explore common association findings for triglyceride levels with and without considering the effect of a drug fenofibrate, as well as adjusting the triglyceride values for high-density lipoprotein (HDL) levels.

## Methods

### Data description

For our analyses, we use pedigree information on triglyceride levels at 4 different time points for 200 nuclear families, along with their genotypes at all the available 597,145 variant sites distributed over 22 autosomal chromosomes, as provided in the Genetics of Lipid Lowering Drugs and Diet Network (GOLDN) data set as part of GAW20. We exclude loci that are monomorphic or have minor allele frequency < 0.05. Because HDL is a potential confounder in the genetic association with triglyceride levels, we use HDL levels at the 4 time points as covariates. To adjust for the effect of fenofibrate, which was administered after the second time point, we perform our transmission disequilibrium analyses based on summarized values of triglyceride and HDL levels before and after the administration of the drug.

### Statistical methodology

#### Imputation of missing values

Data on triglyceride levels and HDL levels are not available for all individuals at every time point. The assumption of multivariate normality provides a computationally elegant framework for the expectation-maximization (EM) algorithm [5] to estimate parameters when data are missing. Because the Kolmogorov-Smirnov test shows significant departure from normality (at level 0.05) for both triglyceride and HDL levels at some of the time points, we perform logarithmic transformations on both phenotypes to induce normality. We use an unrelated set of 117 founders from the pedigrees to estimate the missing phenotype values based on data on the available phenotype values using an EM algorithm as described in Haldar et al. [6]. For the remaining individuals in the pedigree, we use the plug-in parameter estimates of the mean vector and variance–covariance matrix of the phenotypes obtained via the EM algorithm to estimate the missing phenotype values. We then use a generalized linear regression equation of the triglyceride levels on the HDL levels at the 4 time points based on the set of founders to obtain the residuals for all individuals in the pedigree. We use these residuals as phenotype values in our association analyses.

#### Test for transmission disequilibrium using quasi-likelihood

The phenotypes for our association analyses are unadjusted triglyceride levels and triglyceride levels adjusted for HDL levels using the algorithm described in the preceding section. We use a novel quasi-likelihood regression framework based on a resistance generalized estimating equation approach [7] to test for association of a SNP with a multivariate phenotype. For each SNP, we consider all nuclear families in the pedigree with at least 1 heterozygous parent at that SNP. Suppose data are available on *N* nuclear families with *n*_*i*_ offspring in the *i*^th^ family, ***Y***_***j***_ = (*Y*_*j*1_, *Y*_*j*2_, *Y*_*j*3_, …, *Y*_*j*k_) denotes a vector of *k* phenotypes for the *j*^th^ offspring, *Z*_*j*_ and *W*_*j*_ are indicator random variables (1 or 0), respectively denoting whether the heterozygous parent and the other parent (heterozygous or homozygous) at a SNP transmits the minor allele or not to the *j*^th^ offspring. For the *i*^th^ family, we model the conditional distribution of {***X***_***j***_ = (*Z*_*j*_, *W*_*j*_) : *j* = 1, 2, …, *n*_*i*_} given ***Y*** **=** (***Y***_**1**_, ***Y***_**2**_, ***Y***_**3**_, …, ***Y***_***ni***_) using a quasi-likelihood function as follows:

$$ L\left(\alpha, \gamma |X,Y\right)={\Sigma}_{j=1}^{n_i}\left({X}_j-{\lambda}_j\right){V_j}^{-\mathbf{1}}\left({X}_j-{\lambda}_j\right) $$

where ***λ***_j_ (α,γ) is the vector of the conditional expectations of *Z*_*j*_ and *W*_*j*_ given ***Y***_***j***_, both of which are modeled as logistic link functions involving *α* and *γ*, while *V*_*j*_ is the conditional variance–covariance matrix of (*Z*_*j*_,*W*_*j*_) given ***Y***_***j***_. The test for association is equivalent to testing *H*_*0*_*:γ = 0* versus *H*_*1*_*:γ ≠ 0*, and the usual Wald test statistic is distributed as chi-squares with *k* degrees of freedom in the absence of association.

Our association analyses comprise 3 different choices of phenotypes. As fenofibrate was administered after the second time point, we consider the first principal component of the log-transformed triglyceride levels of the first and the second time points along with the first principal component of the log-transformed triglyceride levels of the third and the fourth time points as a bivariate phenotype. We compare the association findings based on this phenotype with (a) the first principal component of the log-transformed triglyceride levels of the first and the second time points (ie, before the administration of fenofibrate) and (b) the first principal component of the log-transformed triglyceride levels of the third and the fourth time points (ie, after the administration of fenofibrate). To evaluate the effect of HDL on triglyceride levels, we perform each of the above analyses for unadjusted log-transformed triglyceride levels and log-transformed triglyceride levels adjusted for HDL levels. We denote the unadjusted bivariate phenotype analysis as MTBAT and the adjusted analysis as MTBATAdj. Similarly, the corresponding univariate analyses based on Kulkarni and Ghosh [3] prior to the administration of fenofibrate are denoted as TBATPre and TBATPreAdj, while those following the administration of the drug are denoted as TBATPost and TBATPostAdj. We, additionally, performed all the test procedures using transmission information only from heterozygous parents (as in the classical transmission disequilibrium test).

## Results

The tests for association are based on 200 nuclear families comprising 990 offspring. As our proposed test procedure requires that at least 1 parent in the family is heterozygous at the marker locus, we selected only those SNPs that have more than 20 informative families. Hence, we performed our analyses on 552,556 SNPs. Of the 990 offspring, data on triglyceride levels were available for 719 offspring at the first time point, 988 offspring at the second time point, 554 offspring at the third time point, and 731 offspring at the fourth time point, and data on HDL levels were available for 719 offspring at the first time point, 989 offspring at the second time point, 709 offspring at the third time point, and 772 offspring at the fourth time point. To correct for multiple testing, we used the Benjamini-Hochberg procedure [8] with an overall false discovery rate (FDR) of 0.05.

The number of SNPs found to be significantly associated with the different phenotype definitions were as follows: 718 based on MTBAT, 685 based on MTBATadj, 147 based on TBATPre, 657 based on TBATPreAdj, 121 based on TBATPost, and 622 based on TBATPostAdj. Among these SNPs, 28 were common for all 6 phenotype definitions, 448 were common between MTBAT and MTBATadj, 80 were common between TBATPre and TBATPreAdj, and 42 were common between TBATPost and TBATPostAdj. With respect to unadjusted triglyceride levels, 51 SNPs were common between MTBAT, TBATPre, and TBATPost, whereas for adjusted triglyceride levels, 390 SNPs were common between MTBATAdj, TBATPreAdj, and TBATPostAdj. The SNPs rs6601447 and rs1986677 located on 8p23.1 within the gene *MSRA*, the SNP rs2466051 located on 8p12 within the gene *NRG1*, the SNP rs1281132 located on 4p16.1 within the gene *SH3TC1*, and multiple SNPs in the region 12q13.12 within the gene *FMNL3* exhibited significant evidence of association with all phenotype definitions. Although the SNP rs1712316 within the gene *SH3TC1* was found to be significantly associated with all phenotype definitions except TBATPreadj, multiple SNPs in the region 10q.24.1 within the gene *ENTPD1* were significantly associated with all phenotype definitions except TBATPost. Among the SNPs mentioned above, rs6601447 and rs1986677 ranked among the top 10 significant findings in all our analyses. We note that although multiple SNPs within the gene *FMNL3* showed significant evidence of association, the significances for the multivariate phenotype were more pronounced (lower *p* values) for the unadjusted triglyceride phenotypes compared to those adjusted for HDL, whereas the opposite phenomenon was observed for the univariate phenotypes, although it seems intuitively difficult to explain the phenomenon. We observe that a higher number of SNPs exhibited significant evidence of association for the bivariate phenotype and that defined by the postdrug measurements compared to the phenotype defined by the predrug measurements. Interestingly, we observed that the analyses based on transmission only from heterozygous parents did not yield a single significant finding with any of the phenotype definitions after FDR correction This is consistent with the results of the simulations corresponding to the quasi-likelihood approach for univariate phenotypes [3] and suggests that transmission information from both parents increases the power of the association tests.

## Discussion and conclusions

In this paper, we modified a transmission-based test for association that includes transmission information from noninformative parents using a quasi-likelihood approach [7] in the multivariate framework. A major advantage of the method is that the retrospective likelihood used to model allelic transmission conditioned on phenotypes does not require any assumptions on the marginal or the joint distributions of phenotype values across different time points.

Many of our association findings are mappable to genes related to heart diseases. The SNP rs2510873, which exhibited significant evidence of association with the bivariate phenotype and was defined by the postdrug measurements, is located in the same genomic region (11q23.3) as the SNP rs964184, which was previously reported to be significantly associated with both triglyceride and HDL levels based on the same GOLDN data [9]. The enzyme *MSRA* (methionine sulfoxide reductase A) protects cardiac myocytes from oxidative stress and is an important therapeutic target for ischemic heart diseases [10]. Neuregulin-1 (*NRG1*) activation improves cardiac function and survival in different forms of cardiomyopathy [11]. The gene *FMNL3* (formin-like 3) is involved in cardiac myofibril development and repair [12]. Because we found that the SNPs located within *FMNL3* exhibited much higher significance with unadjusted triglyceride levels compared to those adjusted for HDL levels, it is likely that the gene modulates the effect of HDL levels rather than the effect of triglyceride levels. We also found that the number of SNPs associated with the bivariate phenotype or the phenotype defined by postdrug measurements was much higher than the phenotype defined by predrug measurements. One possible explanation for this phenomenon is that some of the SNPs modulate the interaction effect of fenofibrate and triglyceride levels. However, separate interaction analyses are necessary to validate this hypothesis.

Linear mixed models are a popular method of choice for genetic association analyses in a family-based framework primarily because such models have the flexibility of accounting for relatedness within families and to correct for population stratification between families [13]. Our proposed quasi-likelihood approach [7] has the implicit assumption that allelic transmissions to the different offspring within a family are uncorrelated, implying that the likelihood is equivalent to that based on independent trios and, hence, the test of association is valid only in the presence of linkage. Moreover, given that the likelihood used in a linear mixed model is prospective in nature, families with both parents homozygous at a SNP can be included in the model and the effect of fenofibrate can be modeled both as a main effect and an interaction effect with SNPs. Such inclusions in the model are likely to yield higher powers of detecting association. On the other hand, the 2 major disadvantages of linear mixed models compared to transmission-based tests are the inherent computational burden involved in analyzing large pedigrees and the susceptibility to violations in distributional assumptions (such as normality). Such violations are particularly common for high-dimensional phenotypes as encountered in longitudinal data, and result in inflated rates of false positives, although simulation studies show that they may be robust to certain model misspecifications [14].

It has been argued that association tests based on imputed phenotypes may lead to biased inferences and it may be more prudent to perform an EM procedure based on the joint likelihood of genotype and phenotype data. However, such a strategy would substantially increase the computational load. Moreover, given that the quasi-likelihood approach is retrospective in nature, the test is less likely to be adversely affected by imputed phenotypes compared to a test based on prospective likelihood. We finally wish to highlight that while the inclusion of transmission information from noninformative parents in the proposed test procedure [7] results in increased power in detecting association, the test is susceptible to inflated false-positive rates in the presence of population stratification.
